# Quantifying Potentially Suitable Geographical Habitat Changes in Chinese Caterpillar Fungus with Enhanced MaxEnt Model

**DOI:** 10.3390/insects16030262

**Published:** 2025-03-03

**Authors:** Yaqin Peng, Danping Xu, Habib Ali, Zhiqian Liu, Zhihang Zhuo

**Affiliations:** 1 College of Life Science, China West Normal University, Nanchong 637002, China; pengyaqin2023@foxmail.com (Y.P.); danpingxu@foxmail.com (D.X.); qnhtvxhp319123@foxmail.com (Z.L.); 2 Department of Agricultural Engineering, Khwaja Fareed University of Engineering and Information Technology, Rahim Yar Khan 64200, Pakistan; habib_ali1417@yahoo.com

**Keywords:** *Ophiocordyceps sinensis*, host insects, Chinese caterpillar fungus, MaxEnt, climate change, suitable habitat

## Abstract

Chinese Caterpillar Fungus (CCF) is a fungal–insect complex formed by the underground larvae of certain species in the family Hepialidae parasitized by *Ophiocordyceps sinensis* (Berk.) (G.H.Sung, J.M.Sung, Hywel-Jones & Spatafora). It is a precious Chinese herbal medicine with significant medicinal value. This study aimed to identify the key environmental factors influencing the distribution of CCFs using the MaxEnt model. First, in the MaxEnt model optimized using the ENMeval package, the most suitable combinations of feature classes and regularization parameters were selected. Second, 22 environmental variables were used to construct distribution models for *O. sinensis*, host insects, and CCFs. Then, the distribution areas of *O. sinensis* and host insects were overlapped to identify highly suitable habitats where both coexist. Finally, these highly suitable habitats were compared to analyze the differences in the distribution areas of *O. sinensis* and host insects and their contributions to the formation of the CCF distribution area. The results showed that elevation, bio18, and bio09 were the primary environmental factors influencing the distributions of *O. sinensis*, host insects, and CCFs. Considering the present, 2050s, and 2070s, the highly suitable areas for all three entities overlapped to a large extent. When we superimposed the high-suitability zones of *O. sinensis* and host insects, the overlapping area was found to be 56.87 × 10^4^ km^2^, which accounted for 5.92% of China’s total land area. The high-suitability area for CCFs was 64.06 × 10^4^ km^2^, accounting for 6.67% of China’s total land area. The findings of this study provide valuable insights into the mechanisms behind the combination of *O. sinensis* and host insects in forming CCFs.

## Simple Summary

Chinese Caterpillar Fungus (CCF) is a precious Chinese herbal medicine formed by the parasitism of *Ophiocordyceps sinensis* (Berk.) (G.H. Sung, J.M. Sung, Hywel-Jones & Spatafora) on the larvae of certain species in the family Hepialidae. In this study, the ENMeval package was used to optimize the MaxEnt model to predict the potential distribution of suitable habitats for *O. sinensis*, host insects, and CCF across China, both in the present and future climate scenarios. The results indicate that elevation, bio18 (precipitation of warmest quarter), and bio09 (mean temperature of driest quarter) are the primary environmental factors influencing their distribution. Considering the present, 2050s, and 2070s, the highly suitable areas for all three entities largely overlap. Additionally, future climate change is expected to somewhat enhance the suitability for their survival. The findings of this study can reveal the current and future potential suitable habitats of Chinese Caterpillar Fungus and contribute to exploring the impact of future environmental changes on the distribution of resources for these species.

## 1. Introduction

Climate plays a crucial role in determining the distribution of species [1]. According to the Fifth Assessment Report (AR5) by the Intergovernmental Panel on Climate Change [2], the global climate is projected to continue warming, with an estimated increase in Earth’s average temperature of 0.3–4.5 °C by the end of the 21st century compared to the period of 1986–2005 [3]. The Qinghai–Tibet Plateau, often referred to as the “Third Pole”, is a unique plateau characterized by high elevation, cold temperatures, and aridity [4]. Since the mid-1950s, the Qinghai–Tibet Plateau has experienced a significantly higher warming trend compared to other regions of China, particularly during the winter season. From 1955 to 1996, the average annual temperature on the plateau rose at a rate of 0.16 °C per decade, while the winter average temperature rose at a rate of 0.32 °C per decade, surpassing the linear warming rate for the same period in the Northern Hemisphere and at similar latitudes [5]. The warming climate has led to permafrost thawing, reduced glacier areas, and increased the risk of natural disasters such as floods, landslides, and the shrinking of wetlands, directly endangering the survival of animals and plants. Moreover, the temperature rise has accelerated the water cycle on the Qinghai–Tibet Plateau, resulting in increased precipitation, which, to some extent, benefits the survival of certain vegetation. However, these changes may prompt organisms that are adapted to the cold and arid climate to migrate to higher latitudes and altitudes [6]. Many studies have examined how animals and plants respond to climate change, but there is limited research on fungi. Chinese Caterpillar Fungus (CCF) is a complex of entomogenous fungi formed by *Ophiocordyceps sinensis* (*O. sinensis*), a member of Clavicipitaceae. This fungi infects the larvae of certain species of moths in the family Hepialidae [7]. The ascospores of *O. sinensis* are found in the soil, and, once they come into contact with the larvae, they parasitize inside them, absorbing nutrients for growth and development. The mycelium then spreads and differentiates, eventually taking over the entire body of the larva and forming a rigid fungal complex, which is called CCF. In winter, the insect–fungus complex takes the form of a rigid insect body, referred to as “winter worm”. By the following spring and summer, as temperatures rise, the fungal mycelium inside the insect breaks through the head of the larva to form a stroma [8], which further grows and emerges above the ground, resembling a sprout of grass. This stage is known as “summer grass”. The host insects of *O. sinensis* have specific habitat requirements and a narrow distribution range. They can only survive in high-altitude, cold areas, mainly on shaded and semi-shaded slopes of mountains at altitudes of 3300–4500 m on the Qinghai–Tibet Plateau.

CCF primarily grows in alpine meadows and alpine shrub meadows that have weak grass cover, calcified soil, and sparse texture [9]. From a global perspective, CCF is found in China, Bhutan, India, and Nepal, but China is its main distribution area. In China, CCF is concentrated in the high-altitude regions of Qinghai, Tibet, Sichuan, Yunnan, and Gansu provinces [10], with Qinghai and Tibet being the primary production areas. CCF is widely recognized for its medicinal and edible value, and it has shown potential antioxidant, anti-tumor, antiviral, and immune-regulating effects. It can help reduce cholesterol, treat low blood pressure, and enhance vascular dilation activity, among other benefits [11]. However, the extensive harvesting of CCFs to meet human demands has led to a significant decline in the population and a severe degradation of the natural habitat. Since 1999, CCF has been classified as a second-level protected endangered species in China. More recently, it has been listed as an endangered species in the 2018 China Biodiversity Red List [7]. In studies that examine the impact of climate change on species’ geographical distributions, species distribution models (SDMs) are widely used in individual ecological research as well as in identifying potential geographic distribution areas for species [12]. These models, which are based on ecological principles and associated algorithms, predict potential regions suitable for species survival and distribution by analyzing known species geographic distributions and corresponding environmental variables [13].

There are several models and software available that can predict species’ potential geographic distributions, including CLIMEX [14], GARP [15], MaxEnt (https://biodiversityinformatics.amnh.org/open_source/maxent/, version 3.4.1, accessed date: 1 July 2023) [16], and the mechanical niche model [17]. Among these methods, the MaxEnt model demonstrates excellent predictive performance and has therefore become one of the most widely applied models. When using the MaxEnt model, only species distribution data and surrounding environmental conditions are required to simulate its ecological niche requirements using maximum entropy theory. This allows for the inference of the species’ potential distribution in the target area [16].

The predictive capability of the MaxEnt model can be enhanced by selecting appropriate model parameters. These parameters include feature combination (FC), regularization multiplier (RM), and maximum background points (BC) [18]. Currently, MaxEnt offers five types of features: linear (L), quadratic (Q), hinge (H), product (P), and threshold (T) [19]. The choice and usage of specific feature combinations depend on the number of species distribution points. In general, the linear feature is always active, the quadratic feature is used when there are more than 10 species distribution points, the hinge feature is employed when there are more than 15 distribution points, and the threshold and product features are utilized when there are more than 80 distribution points [20]. Research suggests that the default settings of the MaxEnt model may not be suitable for predicting the distribution of all species, potentially resulting in overfitting and difficulty in interpreting the final prediction results [21]. Muscarella et al. developed an R package called ENMeval to optimize model parameters. This program assesses model complexity under different parameter conditions by varying the values of the regularization multiplier and feature combination parameters, selecting the model parameters with the lowest complexity for modeling [22].

To investigate the habitat conditions required for *O. sinensis* to infect host insects and form CCFs, as well as the environmental factors influencing its ecological adaptability, we conducted extensive research using geographic reference data and survey materials. The MaxEnt model, optimized with the ENMeval package, was employed to predict the distribution of *O. sinensis* and its host insects in China. The primary objectives of this study are as follows: (1) to forecast the current, 2050s’, and 2070s’ potential distribution of *O. sinensis* and its host insects within China; (2) to predict the current, 2050s’, and 2070s’ potential distribution of CCFs within China; and (3) to explore the key environmental factors influencing the geographic distribution of these species.

## 2. Materials and Methods

### 2.1. Occurrence Collection

**Figure 1:**
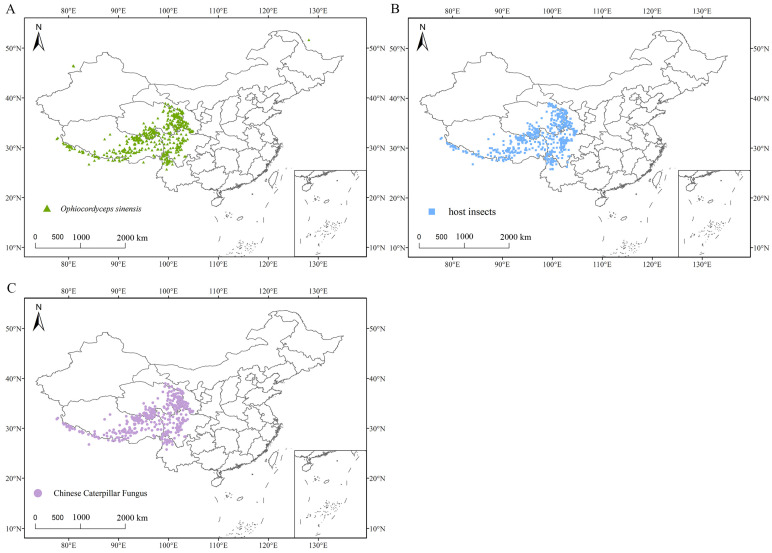
Geographical distribution points of *O. sinensis* (**A**), host insects (**B**), and CCFs (**C**) in China.

The distribution records of these species primarily come from published literature and data from the Global Biodiversity Information Facility (GBIF, https://www.gbif.org/, (accessed on 1 March 2024)). When precise geographic coordinates were lacking in the species distribution records, Google Maps (http://ditu.google.cn/, (accessed on 1 March 2024)) was used to determine longitude and latitude [23]. A total of 490 distribution points for *O. sinensis*, 609 distribution points for host insects, and 424 distribution points for CCFs were obtained. The host insects primarily include *Hepialus armoricanus*, *Hepialus yushuensis* (Chu et Wang, 1985), *Hepialus lagiensis* Yan, *Hepialus biruensis* (Fu, 2002), *Hepialus gonggaensis* (Fu et Huang, 1991), *Hepialus davidi* (Poujade, 1886), *Hepialus menyuanicus* Chu et Wang, 1985, *Thitarodes pui* (Zhang et al.), and *Hepialus xiaojinensis* Tu. To reduce the impact of data duplication and redundancy on the results, distribution points were filtered using ENMTools v1.4 (https://github.com/danlwarren/ENMTools, version 1.4, (accessed on 27 August 2024)) at a spatial resolution of 2.5 arc-minutes (approximately 4.5 km), ensuring that only one distribution point remained per grid cell. Finally, 449 distribution points for *O. sinensis*, 556 distribution points for host insects, and 396 distribution points for CCFs were used to construct the model (Figure 1).

### 2.2. Environment Variables

In this study, 19 bioclimatic variables and 3 terrain factors (elev, slope, and aspect) (Table S1) were used to construct the initial MaxEnt model based on the habitat characteristics of the species. The 19 bioclimatic variables were obtained from the WorldClim global climate database (http://www.worldclim.org, (accessed on 1 March 2024)), covering different periods, including current (1970–2000) and future (2050s and 2070s), with a spatial resolution of 2.5 min. The future climate data were sourced from the BCC-CSM2-MR model within the general circulation model (GCM) [24]. Three scenarios were chosen for this work: SSP1-2.6, SSP3-7.0, and SSP5-8.5. Global Digital Elevation Model (DEM) data were sourced from the National Centers for Environmental Information of the National Oceanic and Atmospheric Administration (NOAA NCEI, https://www.ngdc.noaa.gov/, (accessed on 2 March 2024)), with a spatial resolution of 2.5 arc-minutes [25]. Environmental variables are crucial parameters in constructing niche models, and redundant environmental variables will lead to overfitting, thus reducing the accuracy of the model [13]. To avoid overfitting, multicollinearity among multiple environmental variables was reduced using variance inflation factor (VIF) screening and Pearson correlation. Multicollinearity VIF analysis was performed in SPSS v30 (https://www.ibm.com/cn-zh/spss, (accessed on 1 March 2024)). VIF is the reciprocal of tolerance, and a VIF value less than 10 indicates no multicollinearity among factors; a value between 10 and 100 suggests multicollinearity among factors; and a value greater than 100 indicates serious multicollinearity among factors [26].

In this study, the ENMTools.pl software was used to conduct the correlation analysis of environmental variables, considering variables with Pearson correlation coefficients greater than |0.8| as highly correlated [27]. Compare the contribution rates of these two highly correlated variables in the initial model, and retain the one with the higher contribution rate. Environmental factors with a VIF less than 100 and a correlation less than 0.8 were selected. The remaining environmental variables and species distribution data were imported into the MaxEnt model to calculate contribution rates, removing environmental variables with very low contribution rates [28]. Ultimately, a total of 7, 7, and 6 environmental variables were selected to build models for *O. sinensis*, host insects, and Chinese Caterpillar Fungus (CCF), respectively (Table S2).

### 2.3. Optimization of Model Parameters

In this work, the MaxEnt model was optimized using the ENMeval package in R 3.6.3. The predictive performance of the model primarily depends on the configuration of two key parameters: the regularization multiplier (RM) and the feature combination (FC). Among these, the regularization multiplier significantly influences the distribution characteristics of the prediction results. When the RM value is small, the prediction results tend to exhibit a concentrated and range-restricted distribution pattern. Conversely, a larger RM value leads to prediction results that display a more dispersed and widespread distribution trend [19]. In the optimization process of the MaxEnt model, FC primarily influences the model’s representational capacity and generalization ability [19]. FC and RM work synergistically to jointly affect the model’s complexity and the distribution characteristics of the prediction results. Initially, a block-wise partitioning method was employed to divide the species into four equal parts, with three parts used for training and one part for testing [22]. Subsequently, the Kuenm package in R 3.6.3 (https://www.r-project.org/, (accessed on 3 March 2024)) was used to compare various combinations of the two most crucial parameters (feature class and regularization multiplier) and select the best combination [29]. The MaxEnt model parameters consisted of 5 types of features that can form 31 feature combinations, with the regularization multiplier values ranging from 0.1 to 4 at intervals of 0.1. A total of 1240 candidate models were evaluated, including all combinations of 40 regularization multiplier settings, 31 feature class combinations, and a set of environmental variables [19].

Based on statistical significance (partial receiver operating characteristic, ROC, with 500 iterations), predictive ability (omission rate, OR), and the Akaike information criterion correction (AICc), the function kuenm_ceval was used to optimize model parameters. The final models with the best model parameters were considered significant candidate models based on the “OR_AICc” criterion. These models had omission rates below a threshold (e.g., ≤0.05 in possible scenarios) and the lowest ∆AICc values (≤2) [29].

### 2.4. MaxEnt Modeling

MaxEnt v.3.4.1 (https://biodiversityinformatics.amnh.org/open_source/maxent/, (accessed on 5 March 2024)) was used to establish current and future potential suitable habitat models for *O. sinensis*, host insects, and CCFs. MaxEnt estimates the probability of species presence based on existing records and randomly generates background points by identifying the maximum entropy distribution [30]. Additionally, 75% of occurrences were randomly selected for model training, while the remaining 25% were used for model testing. The running type was set to subsample. The data output format was set as Logistic. The jackknife method was used to evaluate the contribution of environmental variables to habitat selection, and response curves for each environmental factor were plotted. The final output consisted of an average of 10 runs. MaxEnt created a distribution probability raster, with each grid cell value representing the occurrence probability on a scale from 0 to 1 [13]. In ArcGIS v10.8 (ESRI Inc., Redlands, CA, USA), the Jenks’ natural breaks method [25], literature reports, and the actual distribution and habit of the species were used to reclassify suitable habitats for *O. sinensis*, host insects, and CCFs within the Chinese regional scope. These habitats were divided into four categories: unsuitable, low suitability, moderate suitability, and high suitability. The habitat area at different levels was calculated using ArcGIS software, and distribution maps were generated.

### 2.5. Change of Suitable Habitat Distribution Center

The SDM Toolbox, a Python-based GIS toolbox, was used to calculate and compare regional changes in the current and future suitable habitats of species [31]. Using the SDM Toolbox, the centroids of species’ habitats were separately calculated under different emission scenarios for both current and future conditions. In ArcGIS software, the magnitude and direction of the centroid shift of species’ habitat were mapped from the present to the future. *O. sinensis* infects host insects, forming CCFs under favorable environmental conditions. In this study, by overlaying the suitable habitats of *O. sinensis* and host insects, areas suitable for the survival of both were obtained. The centroid movements of both these suitable areas and the CCFs’ suitable areas were mapped. By comparing the centroid movements from both methods, it provided deeper insights into predicting the future distribution changes in CCFs.

### 2.6. Model Evaluation

The accuracy of the MaxEnt model was evaluated using the Area Under the Curve (AUC) value of the ROC. The AUC value ranges from 0 to 1, where AUC < 0.5 indicates random prediction, 0.5 ≤ AUC < 0.7 indicates poor model performance, 0.7 ≤ AUC ≤ 0.9 indicates moderate performance, and AUC > 0.9 indicates high performance [32].

## 3. Results

### 3.1. Model Performance

Among the 1240 candidate models, all exhibited statistical significance. When running the Kuenm package in R 3.6.3, only one model that met both OR and AICc criteria (∆AICc = 0) was obtained. Therefore, the selected MaxEnt model settings for *O. sinensis*, host insects, and CCFs were M_0.8_F_qp_Set1 (regularization multiplier = 0.8, feature class combination = Q and P), M_0.5_F_lpt_Set1 (regularization multiplier = 0.9, feature class combination = L, P, and T), and M_0.8_F_lph_Set1 (regularization multiplier = 0.8, feature class combination = L, P, and H), respectively (Table S3).

The average testing AUC values were 0.978 for *O. sinensis*, 0.976 for host insects, and 0.981 for CCFs (Figure S1). This indicates that these MaxEnt models have high reliability and excellent performance. The MaxEnt software outputs were reclassified using the Reclass tool in ArcGIS to predict the threshold of habitat suitability. Based on the species’ actual habitat suitability and reference literature, habitat suitability was categorized into four levels: unsuitable habitat (0–0.2), low habitat suitability (0.2–0.4), medium habitat suitability (0.4–0.5), and high habitat suitability (0.5–1.0) [33].

### 3.2. Variable Importance

**Figure 2:**
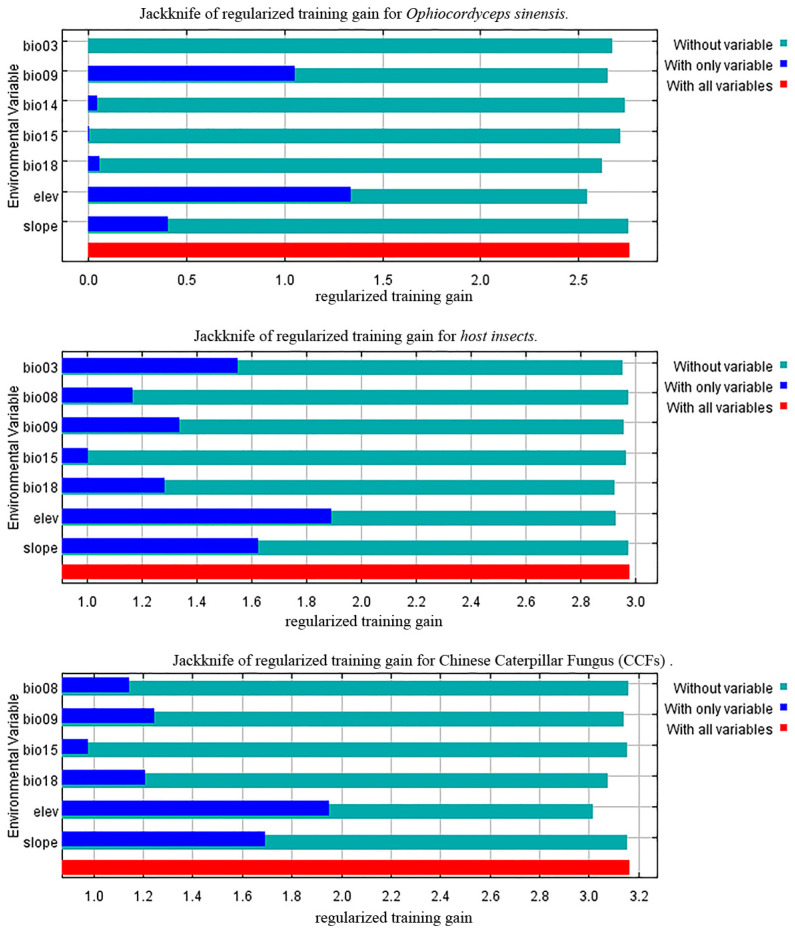
Jackknife test of *O. sinensis* host insects and Chinese Caterpillar Fungus for the environmental variables.

**Figure 3:**
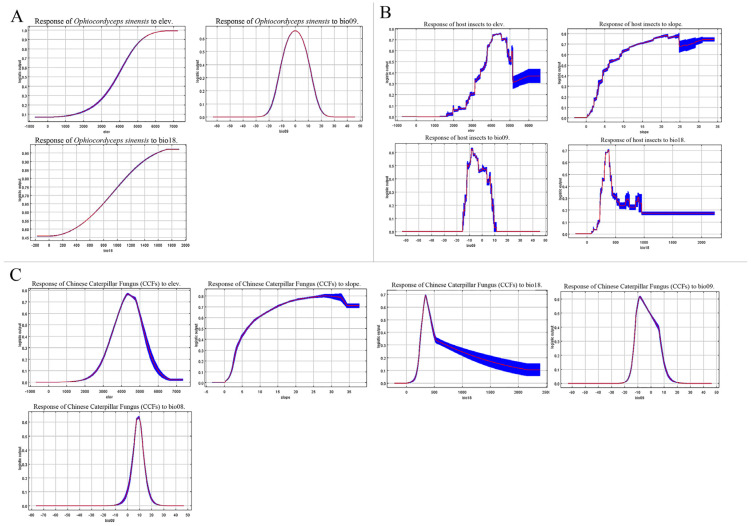
Single response curves of dominant environmental factors of the *O. sinensis* (**A**), host insects (**B**), and CCFs (**C**).

The dominant factors influencing the current potential geographic distribution of these organisms were screened through a comprehensive analysis of climate factors’ contribution rates and jackknife tests. In the MaxEnt model of *O. sinensis*, the environmental factors were ranked in descending order of contribution, with elevation (elev) accounting for 39.6%, followed by the precipitation of warmest quarter (bio18, 28.3%) and the mean temperature of driest quarter (bio09, 15.8%). In the jackknife test, the factors that significantly impacted regularization training gains when using a single climatic factor variable were elevation, slope, bio09 (the mean temperature of driest quarter), bio14 (the precipitation of the driest month), and bio18 (the precipitation of the warmest quarter) (Figure 2). In summary, elevation (elev), bio09, and bio18 were selected for analysis as the three environmental factors that played a key role in the geographic distribution of *O. sinensis* (Table S4). Climatic factors were considered suitable for the species’ survival when the species distribution probability was greater than 0.5. Therefore, the climatic conditions suitable for the survival of *O. sinensis* were as follows: elevation (elev) greater than 3895 m, the mean temperature of the driest quarter (bio09) ranging from −8 °C to 9 °C, and the precipitation of the warmest quarter (bio18) greater than 395 mm (Figure 3A).

In the MaxEnt model of host insects, the main environmental factors, ranked by contribution rate from highest to lowest, were elevation (elev, 43.6%), slope (21.7%), and the precipitation of the warmest quarter (bio18, 17.1%). The jackknife test showed that elevation, slope, bio09, and bio18 significantly impacted regularization training gains (Figure 2). In summary, elev, slope, bio09, and bio18 were the four key environmental factors analyzed for the geographic distribution of host insects (Table S5). The climatic conditions suitable for the survival of host insects were as follows: elevation (elev) ranging from 3731 m to 5159 m, slope greater than 6.1°, the mean temperature of the driest quarter (bio09) between −10 °C and 0 °C, and the precipitation of the warmest quarter (bio18) within the range of 308 mm to 403 mm (Figure 3B).

In the MaxEnt model of CCFs, the main environmental factors, ranked by contribution rate from highest to lowest, were elevation (elev, 49.3%), slope (23.2%), and precipitation of the warmest quarter (bio18, 24%). The jackknife test showed that elev, slope, bio08 (Mean temperature of wettest quarter), bio09 (mean temperature of the driest quarter), and bio18 (precipitation of the warmest quarter) significantly impacted regularization training gains (Figure 2). In summary, elev, slope, bio08, bio09, and bio18 were the five key environmental factors analyzed for the geographic distribution of CCFs (Table S6). The climatic conditions suitable for the survival of CCFs were as follows: elevation (elev) ranging from 3619 m to 5164 m, slope greater than 6.75°, mean temperature of the wettest quarter (bio08) between 6.5 °C and 11.75 °C, mean temperature of the driest quarter (bio09) between −10.5 °C and −1 °C, and precipitation of the warmest quarter (bio18) within the range of 288 mm to 460 mm (Figure 3C).

### 3.3. The Potential Suitable Habitat Under Current Climate Conditions

The potential geographic distribution areas of *O. sinensis*, host insects, and CCFs under current climatic conditions were analyzed using MaxEnt. Spatial analysis methods in ArcGIS were employed to statistically analyze the suitable habitat areas for each level. The results showed that the high-suitability habitat area for *O. sinensis* was 74.28 × 10^4^ km^2^, the moderate-suitability habitat area was 25.19 × 10^4^ km^2^, the low-suitability habitat area was 93.75 × 10^4^ km^2^, and the unsuitable habitat area was 767.85 × 10^4^ km^2^. The high-suitability habitat accounted for 7.73% of the total national area, while the moderate-suitability habitat accounted for 2.62% (Table S7). The high-suitable habitat was mainly located in the southeastern part of the Qinghai–Tibet Plateau, Hengduan Mountains, and their surrounding areas (Figure S2). The statistical data for the highly suitable areas of *O. sinensis* are presented in Table S8. The results indicate that the highly suitable distribution areas are mainly concentrated in the provinces of Gansu, Qinghai, Sichuan, the Tibet Autonomous Region, and Yunnan. These areas account for 0.28%, 1.12%, 3.79%, 2.37%, and 0.16% of the total national area, respectively. The highly suitable area in the Tibet Autonomous Region is particularly large, covering 36.47 × 10^4^ km^2^, which is 29.69% of the province’s total area. Sichuan province has the second-largest highly suitable area, covering 22.80 × 10^4^ km^2^, which is 46.91% of the province’s total area.

The high-suitability habitat area for host insects is 66.63 × 10^4^ km^2^, the moderate-suitability habitat area is 36.25 × 10^4^ km^2^, the low-suitability habitat area is 47.48 × 10^4^ km^2^, and the unsuitable habitat area is 810.70 × 10^4^ km^2^. The high-suitability habitat area accounts for 6.93% of the total national area, while the moderate-suitability habitat area accounts for 3.77% (Table S7). The high-suitable habitat primarily occurs in the southeastern part of the Qinghai–Tibet Plateau, the southeastern regions of the Qilian Mountains, and along the Hengduan Mountains (Figure S3).

The statistical data for the highly suitable distribution areas of host insects are shown in Table S9. The results indicate that the highly suitable distribution areas are mainly concentrated in the provinces of Gansu, Qinghai, the Tibet Autonomous Region, Sichuan, and Yunnan, accounting for 0.38%, 1.29%, 2.67%, 2.28%, and 0.30% of the total national area, respectively. Specifically, the highly suitable area in the Tibet Autonomous Region is the largest, covering 25.71 × 10^4^ km^2^, which is 20.93% of the province’s total area. Sichuan province has the second-largest highly suitable area, covering 21.95 × 10^4^ km^2^, which is 45.17% of the province’s total area.

**Figure 4:**
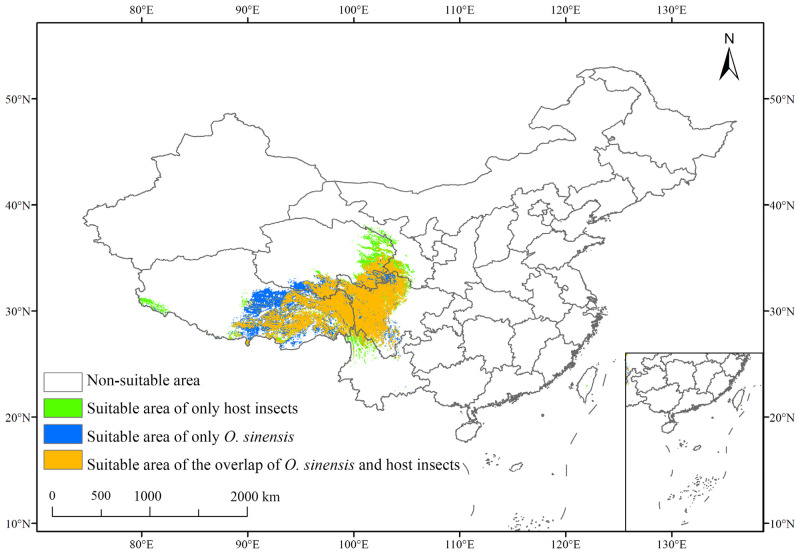
Distribution of suitable habitats for both *O. sinensis* and host insects to coexist under the current climate.

By overlaying the potential suitable habitat of *O. sinensis* with that of its host insects, we obtained the following areas: potential suitable areas where both *O. sinensis* and host insects can thrive, potential suitable areas where only *O. sinensis* can thrive, potential suitable areas where only host insects can thrive, and non-habitat areas. The area suitable for both *O. sinensis* and host insects to coexist is 56.87 × 10^4^ km^2^ and the non-suitable area is 877.03 × 10^4^ km^2^. The area suitable for both *O. sinensis* and host insects to coexist accounts for 7.08% of the total area of the country (Table S7), primarily distributed in the southeastern part of the Qinghai–Tibet Plateau and along the Hengduan Mountains (Figure 4).

The statistical data for the coexistence of *O. sinensis* and host insects in suitable areas were presented in Table 1. The results showed that these areas were primarily concentrated in Gansu province, Qinghai province, Tibet Autonomous Region, Sichuan province, and Yunnan province. Specifically, they accounted for 0.24%, 0.90%, 2.45%, 2.19%, and 0.14% of the country’s total area, respectively. Among these regions, Tibet Autonomous Region had the largest highly suitable area, covering 23.51 × 10^4^ km^2^, which accounted for 19.13% of the province’s total area. Sichuan province had the second-largest highly suitable area, covering 21.09 × 10^4^ km^2^, which accounted for 43.39% of the province’s total area.

**Figure 5:**
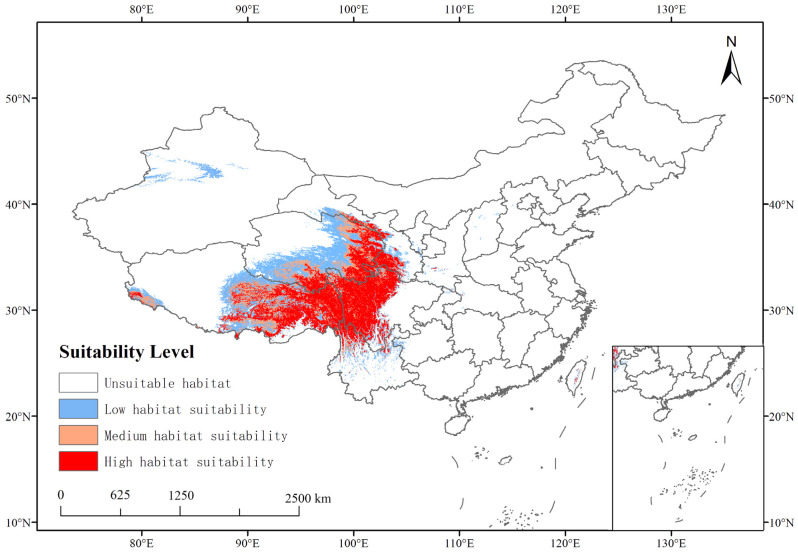
Distribution of suitable habitats for CCFs under the current climate.

The area highly suitable for CCFs was 64.06 × 10^4^ km^2^, the area moderately suitable was 29.28 × 10^4^ km^2^, the area with low suitability was 52.39 × 10^4^ km^2^, and the unsuitable habitat area was 815.35 × 10^4^ km^2^. The highly suitable habitat accounted for 6.67% of the total national area, while the moderately suitable habitat accounted for 3.05% (Table S7). The highly suitable habitat was primarily found in the southeastern part of the Qinghai–Tibet Plateau, the Qilian Mountains, and the Hengduan Mountains, ranging from 25° N to 39° N and 87° E to 105° E (Figure 5).

The statistical data regarding the distribution areas of CCFs, which are highly suitable, are presented in Table 2. The results demonstrate that, in theory, the distribution areas of CCFs are primarily concentrated in Gansu province, Qinghai province, the Tibet Autonomous Region, Sichuan province, Yunnan province, and Taiwan province. These areas account for 0.46%, 1.21%, 2.47%, 2.21%, 0.31%, and 0.01% of the total national area, respectively. The largest highly suitable area is found in the Tibet Autonomous Region, covering 23.71 × 10^4^ km^2^, which accounts for 19.30% of the province’s total area. Sichuan province has the second-largest highly suitable area, covering 21.27 × 10^4^ km^2^, accounting for 43.77% of the province’s total area.

By comparing the distribution ranges of these species, we can observe that the southeastern part of the Qilian Mountains was moderately suitable for *O. sinensis* but highly suitable for host insects. The southern part of the central Qinghai–Tibet Plateau was suitable for the survival of *O. sinensis* but not for host insects. The western part of the Himalayas was more suitable for host insects. Overall, the distribution area suitable for both *O. sinensis* and host insects highly overlapped with the current distribution area of CCFs. Therefore, to a large extent, CCFs can be formed in areas suitable for the coexistence of *O. sinensis* and host insects.

### 3.4. Potential Changes in Suitable Habitats Under Future Climate Conditions

**Figure 6:**
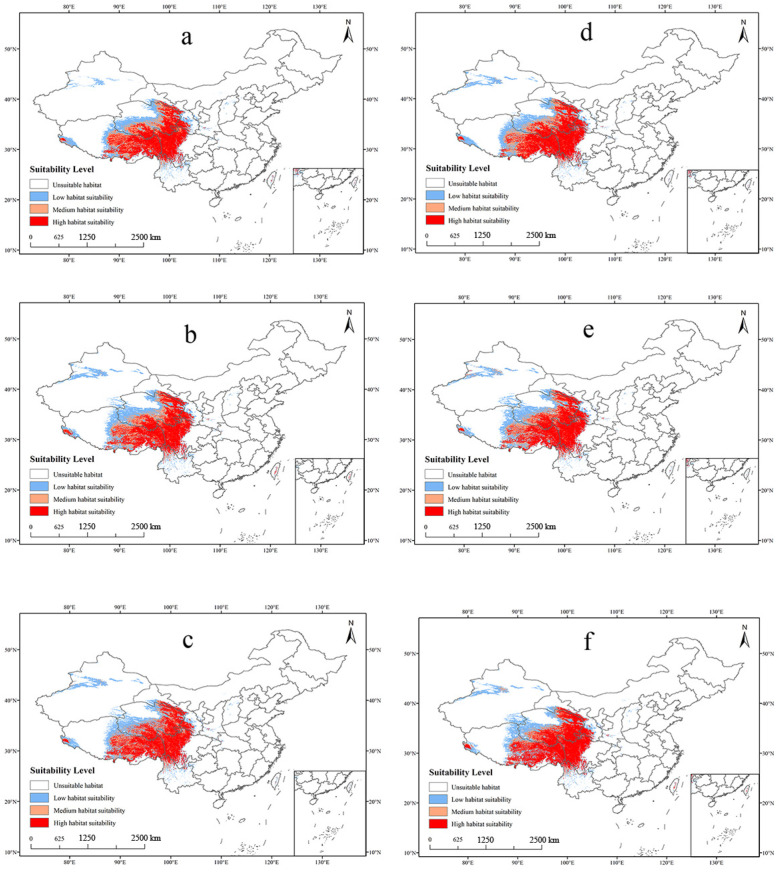
Potential distribution of CCFs based on different future climate scenarios. (**a**) 2050s-SSP1-2.6; (**b**) 2050s-SSP3-7.0; (**c**) 2050s-SSP5-8.5; (**d**) 2070s-SSP1-2.6; (**e**) 2070s-SSP3-7.0; (**f**) 2070s-SSP5-8.5).

**Figure 7:**
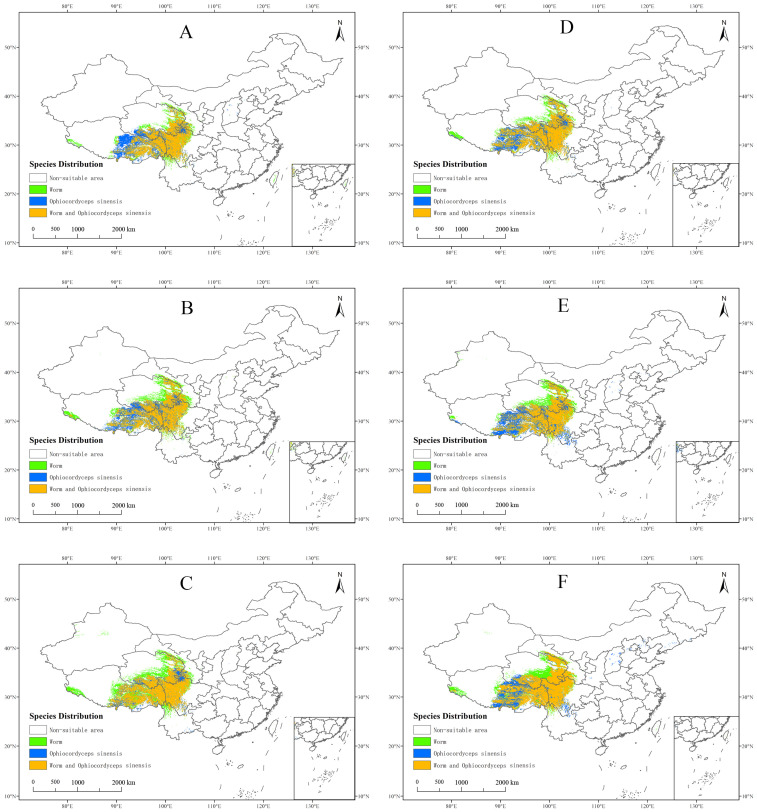
Potential distribution of areas where *O. sinensis* and host insects can coexist based on different future climate scenarios. (**A**) 2050s-SSP1-2.6; (**B**) 2050s-SSP3-7.0; (**C**) 2050s-SSP5-8.5; (**D**) 2070s-SSP1-2.6; (**E**) 2070s-SSP3-7.0; (**F**) 2070s-SSP5-8.5.

In this study, the potential distribution of *O. sinensis* (Figure S4), host insects (Figure S5), and CCFs (Figure 6) was predicted for the 2050s and 2070s using the optimized MaxEnt model under three different greenhouse gas emission pathways: SSP1-2.6, SSP3-7.0, and SSP5-8.5. The areas with high suitability for *O. sinensis*, host insects, and CCFs in the 2050s and 2070s were mainly located in the southeastern Qilian Mountains, the southeastern Bayan Har Mountains, the Hengduan Mountains, the eastern part of the Qinghai–Tibet Plateau, and the northwestern and southeastern parts of the Himalayas. These highly suitable distribution areas largely overlapped (Figure 6 and Figure 7).

Under the three scenarios of SSP1-2.6, SSP3-7.0, and SSP5-8.5, the distribution area of highly suitable habitat for *O. sinensis* varied significantly from the present to the 2070s, demonstrating an overall increasing trend (Table S10). In scenario SSP5-8.5 of the 2070s, the distribution area of highly suitable habitat for *O. sinensis* experienced the greatest increase, amounting to 32.29%. From the present to the 2070s, the area of highly suitable habitat for host insects underwent both an increase and subsequent decrease. In scenario SSP5-8.5 for the 2050s, the distribution area of highly suitable habitat for host insects demonstrated the highest increase, reaching 47.06% (Table S11).

The changes in the area suitable for the coexistence of *O. sinensis* and host insects from the present to the 2070s mirrored the changes in the distribution of *O. sinensis*’ highly suitable habitat. In scenario SSP5-8.5 for the 2070s, this highly suitable habitat area experienced the greatest increase, totaling 40.12% (Table 3).

From the present to the 2070s, according to the SSP5-8.5 scenario, the changes in the area highly suitable for the survival of CCFs were found to be similar to those in the area suitable for the coexistence of *O. sinensis* and host insects, with an increase of 44.78% (Table 4). Looking ahead, under the three emission scenarios in the future, the trends in the changes in the area of highly suitable habitat for *O. sinensis*, host insects, and CCFs indicate their adaptability to future climate change.

### 3.5. Shift of Appropriate Habitat Distribution Center

**Figure 8:**
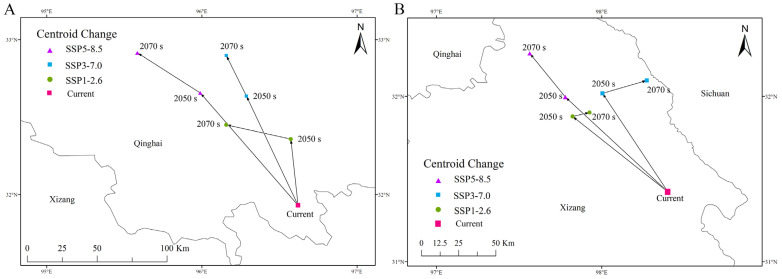
Shift in the centroids of highly suitable habitats of both *O. sinensis* and host insects to coexist (**A**) and CCFs (**B**) under three future climate scenarios.

In this study, we used ArcGIS tools to overlay the highly suitable habitats of *O. sinensis* and host insects. This allowed us to identify areas where *O. sinensis* and host insects can coexist. The results showed that the centroid of the suitable habitat area, where *O. sinensis* and host insects coexist, is located in Nangqian County, Yushu Tibetan Autonomous Prefecture, Qinghai province (96°37’10” E 31°55’52” N) (Figure 8A). According to our projections, by the 2070s, the centroid will shift to different locations depending on the scenarios: 96°9’25” E 32°27’3” N under the SSP1-2.6 scenario, 96°9’25” E 32°53’51” N under the SSP3-7.0 scenario, and 95°35’5” E 32°54’54” N under the SSP5-8.5 scenario. Overall, the centroid of the highly suitable habitat area for *O. sinensis* is moving towards the northwest.

Currently, the distribution center of the CCF’s suitable habitat is located in Chamdo City, Tibet Autonomous Region (98°24′4″ E, 31°25′14″ N) (Figure 8B). By the 2090s, according to the SSP1-2.6, SSP2-4.5, SSP3-7.0, and SSP5-8.5 scenarios, the distribution center is projected to shift to 97°55′38″ E 31°54′9″ N, 98°17′14″ E 31°56′52″ N, and 97°33′56″ E 32°15′37″ N, respectively. Overall, the movement range of the CCF’s centroid is minimal, with the suitable habitat shifting in the northwest direction. In summary, the centroids of the suitable habitats for both species move in the same direction, shifting towards the northwest and higher latitudes over time.

## 4. Discussion

Previous studies often used default parameters in MaxEnt models to predict species distribution, which could lead to overfitting and sampling bias, thus affecting the accuracy of predictions. In this study, we utilized the ENMeval package in R 3.6.3 to optimize model parameters. As a result, all three MaxEnt AICc values decreased to 0, indicating reduced overfitting after optimization [26]. The optimized AUC values were all above 0.9, indicating high accuracy in the results.

Based on current climatic conditions, the potential suitable distribution areas for CCFs were mainly located in the southeastern part of Gansu, the southeastern part of Qinghai, the eastern part of Tibet, the western part of Sichuan, and the northwestern part of Yunnan. These findings are consistent with the collection points of CCFs and previous predictive results [7,34,35].

The distribution of CCFs is closely related to the distribution of *O. sinensis* and its host. The MaxEnt model’s jackknife test revealed that elevation, the precipitation of the warmest quarter (bio18), and the mean temperature of the driest quarter (bio09) played a major role in the distribution of *O. sinensis*, its host, and CCFs. This highlights the inseparable relationship between the distribution of CCFs and elevation, precipitation, and temperature. According to the response curves of environmental factors, CCFs were found to thrive in high-altitude, cold, and humid areas. These findings align with previous studies, indicating that CCFs are typically found in high-altitude and cold regions [36].

Elevation affects temperature and precipitation, with temperature showing a negative correlation with increasing elevation. There is a complex relationship between elevation and precipitation [37]. The aspect of a mountain slope also influences precipitation, with windward slopes receiving more precipitation than leeward slopes [38]. Therefore, altitude further influences the richness of the community, often resulting in different plant communities in different altitude regions. High-altitude areas often feature vegetation types such as coniferous forests, mixed coniferous and broad-leaved forests, shrubs, and alpine meadows [39]. Host insects are mostly distributed in alpine meadows and shrub areas, which may be related to their feeding habits. Host insects primarily feed on the roots and stems of plants such as Polygonum viviparum, Polygonum capitatum, Polygonum macrophyllum, and Rheum pumilum, as well as some species of the Fabaceae family like Astragalus membranaceus. These plants often form meadows or shrubs [40]. Precipitation and temperature are also factors that affect the growth and reproduction of *O. sinensis* and host insects. Fungi typically inhabit damp, dark environments that are rich in humus and have relatively high air humidity [41]. Environmental humidity directly affects the moisture content inside host insects and can disrupt the water balance within their bodies, thus impacting individual development [42]. Temperature can influence the number of adult insects that emerge and their egg production. In unsuitable temperature conditions, the reproductive capacity of insects weakens.

CCFs have a strong regional distribution, primarily occurring in the plateau and mountainous regions of southwestern China [43]. The warmest quarter precipitation (bio18) is an important environmental variable that affects the distribution of CCFs. As the warmest quarter precipitation increases, the suitability of CCFs first rises and then decreases, exhibiting a “convex” shape. The trend in the distribution of suitable habitats for host insects follows a similar pattern to that of CCFs, but the suitable habitat for *O. sinensis* increases until it reaches its maximum value. This phenomenon may reveal that the changes in suitable habitats for CCFs correspond to the changes in suitable habitats for host insects.

The most suitable mean temperatures of the driest quarter (bio9) for the survival of *O. sinensis*, host insects, and CCFs are 0.5 °C, −7.2 °C, and −8.2 °C, respectively, and the trends in their suitable habitats align with changes in temperature. All the suitable environmental factors for the growth of CCFs are within this range.

In different current and future climate scenarios, the most suitable habitat distribution for CCFs is located in the southeastern part of Gansu, the southeastern part of Qinghai, the eastern part of Tibet, the western part of Sichuan, and the northwestern part of Yunnan. A highly suitable habitat for a species is considered the core area of its germplasm distribution, which exhibits genetic diversity [25]. Therefore, it is necessary to strengthen the conservation of germplasm resources for CCFs in these areas. Under different climate scenarios, the potential distribution area of Chinese Caterpillar Fungus (CCF) is generally increasing. By comparing the distribution areas of *O. sinensis* (the fungus) with those of the host insects, we have identified the areas where both species coexist. Specifically, under the SSP5-8.5 scenario, the increase in the co-distribution area of *O. sinensis* and host insects is similar to the change in the distribution area of CCFs. Using future climate conditions, we have observed that the future centroids of both species are moving towards higher latitudes and altitudes. This shift towards higher altitudes and latitudes is a trend observed in many species due to global climate warming [44]. In the 2070s, under the SSP5-8.5 scenario, we found that the distribution of CCFs shows the most significant growth. This indicates that under future conditions of increased temperature and precipitation, the potential distribution area of CCFs may increase. Growing conditions within a suitable range are beneficial to plant growth and provide more food resources for host insects. Higher temperatures can accelerate the reproduction of *O. sinensis* and the development of host insects.

Shrestha et al. discovered that the future potential distribution of suitable habitats for CCFs exhibits an increasing trend, but the direction of expansion generally shifts towards the northeast. This may be attributed to their failure to optimize the model and their selection of the Himalayan region as the study area [43]. The potential suitable habitat areas currently output by the CCFs model, which were not optimized by Jie et al., are generally consistent with the findings of this study. However, the suitable habitat area predicted by them is smaller than ours. This discrepancy may be attributed, on one hand, to the smaller amount of species distribution data they incorporated and, on the other hand, to slight differences in the threshold settings used for delineating suitable habitat areas [35]. The formation mechanism of CCFs is complex and dependent on various factors, including soil physicochemical properties, vegetation, light exposure, and human activities. Human interference can have a significant impact on the distribution of CCFs. Soil structure and vegetation resources can be disrupted, leading to the loss of suitable microhabitats for CCFs [7]. CCFs are sensitive to external pollution and have a limited suitable habitat area. In recent years, due to increased human activities and a growing demand for CCFs, the continuous exploitation of their resources has been carried out. This excessive exploitation not only decreases the number of CCFs but also disrupts the living environment of *O. sinensis* and host insects, resulting in a decline in the quantity and quality of CCFs in specific locations. Therefore, it is crucial to accurately define the natural distribution areas of CCFs and prioritize their protection. By implementing sustainable harvesting practices, we can develop and utilize the resources of CCFs in a responsible manner.

## 5. Conclusions

Based on current and future climate change scenarios, we employed the parameter-optimized MaxEnt model for the first time to simulate the potential suitable distribution areas of *O. sinensis*, host insects, and Chinese Caterpillar Fungus (CCFs). We overlaid the habitat ranges of *O. sinensis* and host insects to identify areas suitable for the survival of both species. By combining this region with the potential suitable distribution areas of CCFs, our analysis indicated that, under current climatic conditions, CCFs were primarily distributed in high-altitude areas in southwestern China. The high-suitability areas were mainly located in the southeastern part of Gansu, the southeastern part of Qinghai, the eastern part of Tibet, the western part of Sichuan, and the northwestern part of Yunnan, ranging from 25° N to 39° N and 87° E to 105° E. Through MaxEnt prediction, we identified several important environmental variables that affect the distribution of CCFs, including elevation, slope, bio18 (precipitation of the warmest quarter), and bio9 (mean temperature of the driest quarter). Under global climate change conditions, the potential suitable area for CCFs is expected to increase, with the expansion rate of high-suitability areas ranging from 5.69% to 45.34%. The predicted centroids of potential high-suitability areas are projected to move northwestward.

## Figures and Tables

**Table 1:** Analysis of the highly suitable distribution areas for both *O. sinensis* and host insects to coexist.

Province	Highly Suitable Area (10^4^ km^2^)	Total (10^4^ km^2^)	Percentage of Highly Suitable Area in Province (%)	Percentage of Highly Suitable Areas in China (%)
Gansu	2.29	42.59	5.37	0.24
Qinghai	8.62	72.1	11.95	0.90
Tibet	23.51	122.84	19.13	2.45
Sichuan	21.09	48.6	43.39	2.19
Yunnan	1.37	39.4	3.48	0.14
China	56.87	/	/	5.92

**Table 2:** Analysis of the highly suitable distribution areas for CCFs.

Province	Highly Suitable Area (10^4^ km^2^)	Total (10^4^ km^2^)	Percentage of Highly Suitable Area in Province (%)	Percentage of Highly Suitable Areas in China (%)
Gansu	4.39	42.59	10.30	0.46
Qinghai	11.65	72.1	16.15	1.21
Tibet	23.71	122.84	19.30	2.47
Sichuan	21.27	48.6	43.77	2.21
Yunnan	2.95	39.4	7.48	0.31
Shanxi	0.04	20.56	0.19	-
Taiwan	0.05	3.60	1.49	0.01
China	64.06	/	/	6.67

**Table 3:** Areas of suitable habitats for both *O. sinensis* and host insects to coexist under future climate scenarios.

Decade Scenarios	Predicted Area (×10^4^ km^2^)	The Percentage Relative to the Total Area of China	Comparison with Current Distribution (%)
	High Habitat Suitability	High Habitat Suitability	High Habitat Suitability
Current	56.87	5.92	
2050s-SSP1-2.6	59.21	6.16	4.12
2050s-SSP3-7.0	62.61	6.52	10.11
2050s-SSP5-8.5	70.63	7.35	24.20
2070s-SSP1-2.6	65.57	6.82	15.30
2070s-SSP3-7.0	63.19	6.58	11.13
2070s-SSP5-8.5	79.68	8.29	40.12

**Table 4:** Areas of suitable habitats for CCFs under future climate scenarios.

Decade Scenarios	Predicted Area (×10^4^ km^2^)				Total Suitability Area as a Percentage of China’s Total Area	Comparison with Current Distribution (%)			
	Low Habitat Suitability	Medium Habitat Suitability	High Habitat Suitability	Total Suitability Area		Low Habitat Suitability	Medium Habitat Suitability	High Habitat Suitability	Total Suitability Area
Current	52.39	29.28	64.06	145.72	15.16				
2050s-SSP1-2.6	47.56	34.13	77.00	158.69	16.59	−9.20%	16.59%	20.20%	8.91
2050s-SSP3-7.0	58.09	35.61	77.02	170.72	16.27	10.89%	21.63%	20.24%	17.16
2050s-SSP5-8.5	57.41	32.31	79.26	168.99	15.74	9.59%	10.38%	23.74%	15.97
2070s-SSP1-2.6	51.93	33.40	80.62	165.95	16.31	−0.87%	14.10%	25.86%	13.88
2070s-SSP3-7.0	57.43	32.47	78.00	167.90	16.51	9.62%	10.91%	21.77%	15.22
2070s-SSP5-8.5	56.24	25.66	92.74	174.64	16.16	7.35%	−12.33%	44.78%	19.85

## Data Availability

The data supporting the results are available in a public repository at GBIF.org (1 March 2024) GBIF Occurrence Download https://doi.org/10.15468/dl.rsuphw, accessed on 1 March 2024 and Yaqin Peng (2024): *Ophiocordyceps sinensis* occurrence.xlsx. figshare. Dataset. https://doi.org/10.6084/m9.figshare.25323250.v1, accessed on 1 March 2024.

## Supplementary Materials

The following supporting information can be downloaded at https://www.mdpi.com/article/10.3390/insects16030262/s1, Figure S1: Ophiocordyceps sinensis’s Host insects’ and Chinese Caterpillar Fungus’ ROC curve and the values of AUC for the optimized MaxEnt model.; Figure S2: Distribution of suitable habitats for *O. sinensis* under the current climate.; Figure S3: Distribution of suitable habitats for host insects under the current climate.; Figure S4: Potential distribution of *O. sinensis* based on different future climate scenarios. (a), 2050s-SSP126; (b), 2050s-SSP370; (c), 2050s-SSP585; (d), 2070s-SSP126; (e), 2070s-SSP370; (f), 2070s-SSP585. Figure S5: Potential distribution of host insects based on different future climate scenarios. (a), 2050s-SSP126; (b), 2050s-SSP370; (c), 2050s-SSP585; (d), 2070s-SSP126; (e), 2070s-SSP370; (f), 2070s-SSP585. Table S1: Environmental variables related to the distributions; Table S2: The environmental variables used in this study; Table S3: The optimize model parameters; Table S4: Percentage contribution of 7 environment variables of the *O. sinensis*; Table S5: Percentage contribution of 7 environment variables of host insects; Table S6: Percentage contribution of 6 environment variables of the Chinese Caterpillar Fungus (CCFs); Table S7: Current areas of habitat distribution for *Ophiocordyceps sinensis*, host insects, and the Chinese Caterpillar Fungus; Table S8: Analysis of the highly suitable distribution areas for *O. sinensis*; Table S9: Analysis of the highly suitable distribution areas for host insects; Table S10: Areas of suitable habitats for *O. sinensis* under future climate scenarios; Table S11: Areas of suitable habitats for host insets under future climate scenarios.

## Abbreviations

The following abbreviations are used in this manuscript:

CCFs	Chinese Caterpillar Fungus
SDMs	species distribution models
*O. sinensis*	*Ophiocordyceps sinensis*
