# Supplementary material for: Quantifying Potentially Suitable Geographical Habitat Changes in Chinese Caterpillar Fungus with Enhanced MaxEnt Model
Source: Insects. 2025 Mar 3;16(3):262. doi: 10.3390/insects16030262 (PMC11943047; doi:10.3390/insects16030262)
Supplement: Supplementary file 1 [file insects-16-00262-s001.zip › Supplementary Table S1.pdf]

Table S1 Environmental variables related to the distributions.

Abbreviation	Climate variables	Unit
Bio1	Annual mean temperature	℃
Bio2	Mean diurnal temperature range	℃
Bio3	Isothermality (bio2 / bio7) (× 100)	
Bio4	Temperature Seasonalit (standard deviation×100)	
Bio5	Max temperature of warmest month	℃
Bio6	Min temperature of coldest month	℃
Bio7	Temperature annual range (bio5- bio6)	℃
Bio8	Mean temperature of wettest quarter	℃
Bio9	Mean temperature of driest quarter	℃
Bio10	Mean temperature of warmest quarter	℃
Bio11	Mean temperature of coldest quarter	℃
Bio12	Annual precipitation	mm
Bio13	Precipitation of wettest month	mm
Bio14	Precipitation of driest month	mm
Bio15	Precipitation seasonality (Coefficient of variation)	
Bio16	Precipitation of wettest quarter	mm
Bio17	Precipitation of driest quarter	mm
Bio18	Precipitation of warmest quarter	mm
Bio19	Precipitation of coldest quarter	mm
Elev	Altitude (elevation above sea level) (m)	m
Slope	Slope	°
Aspect	Aspect	rad
