# Supplementary material for: Quantifying Potentially Suitable Geographical Habitat Changes in Chinese Caterpillar Fungus with Enhanced MaxEnt Model
Source: Insects. 2025 Mar 3;16(3):262. doi: 10.3390/insects16030262 (PMC11943047; doi:10.3390/insects16030262)
Supplement: Supplementary file 1 [file insects-16-00262-s001.zip › Supplementary Table S10.pdf]

Table S10 Areas of suitable habitats for *O. sinensis* under future climate scenarios.

Decade Scenarios	Predicted Area（$\times10^4$ km^2）				Comparison with Current Distribution（%）			
	Low Habitat Suitability	Medium Habitat Suitability	High Habitat Suitability	Total Area	Low Habitat Suitability	Medium Habitat Suitability	High Habitat Suitability	Total Area
Current	93.75	25.19	74.28	193.22				
2050s-SSP1-2.6	104.97	30.41	81.63	217.01	11.96	20.76	9.90	12.32
2050s-SSP3-7.0	86.95	25.47	82.71	195.14	-7.25	1.12	11.36	0.99
2050s-SSP5-8.5	95.51	27.50	80.69	203.69	1.87	9.17	8.63	5.42
2070s-SSP1-2.6	109.03	25.61	84.33	218.96	16.30	1.67	13.53	13.33
2070s-SSP3-7.0	109.05	27.75	88.01	224.81	16.31	10.18	18.49	16.35
2070s-SSP5-8.5	135.62	27.11	98.26	260.99	44.65	7.64	32.29	35.08
