# Supplementary material for: Quantifying Potentially Suitable Geographical Habitat Changes in Chinese Caterpillar Fungus with Enhanced MaxEnt Model
Source: Insects. 2025 Mar 3;16(3):262. doi: 10.3390/insects16030262 (PMC11943047; doi:10.3390/insects16030262)
Supplement: Supplementary file 1 [file insects-16-00262-s001.zip › Supplementary Table S11.pdf]

Table S11 Areas of suitable habitats for host insets under future climate scenarios.

Decade Scenarios	Predicted Area (×km^2)				Comparison with Current Distribution (%)			
	Low Habitat Suitability	Medium Habitat Suitability	High Habitat Suitability	Total Area	Low Habitat Suitability	Medium Habitat Suitability	High Habitat Suitability	Total Area
Current	47.48	36.25	66.63	150.36				
2050s-SSP1-2.6	53.54	45.92	70.42	169.88	12.75	26.67	5.69	12.98
2050s-SSP3-7.0	58.98	46.78	80.79	186.55	24.21	29.05	21.25	24.07
2050s-SSP5-8.5	44.78	37.95	97.98	180.72	-5.68	4.69	47.06	20.19
2070s-SSP1-2.6	52.06	41.31	77.78	171.14	9.63	13.94	16.73	13.82
2070s-SSP3-7.0	70.24	42.16	77.39	189.79	47.94	16.30	16.15	26.22
2070s-SSP5-8.5	50.04	37.79	96.84	184.67	5.39	4.24	45.34	22.82
