# Supplementary material for: Quantifying Potentially Suitable Geographical Habitat Changes in Chinese Caterpillar Fungus with Enhanced MaxEnt Model
Source: Insects. 2025 Mar 3;16(3):262. doi: 10.3390/insects16030262 (PMC11943047; doi:10.3390/insects16030262)
Supplement: Supplementary file 1 [file insects-16-00262-s001.zip › Supplementary Table S2.pdf]

**Table S2 The environmental variables used in this study.**

| Species | Abbreviation | Climate variables | Unit |
|---|---|---|---|
| *Ophiocordyceps sinensis*、host insects and Chinese Caterpillar Fungus | Bio3 | Isothermality (bio2 / bio7) (× 100) | |
| host insects and Chinese Caterpillar Fungus | Bio8 | Mean temperature of wettest quarter | ℃ |
| *Ophiocordyceps sinensis*、host insects and Chinese Caterpillar Fungus | Bio9 | Mean temperature of driest quarter | ℃ |
| *Ophiocordyceps sinensis*、 | Bio14 | Precipitation of driest month | mm |
| *Ophiocordyceps sinensis*、host insects and Chinese Caterpillar Fungus | Bio15 | Precipitation seasonality (Coefficient of variation) | |
| *Ophiocordyceps sinensis*、host insects and Chinese Caterpillar Fungus | Bio18 | Precipitation of warmest quarter | mm |
| *Ophiocordyceps sinensis* and host insects | Elev | Altitude (elevation above sea level) (m) | m |
| *Ophiocordyceps sinensis* and host insects | Slope | Slope | ° |
