# Supplementary material for: Quantifying Potentially Suitable Geographical Habitat Changes in Chinese Caterpillar Fungus with Enhanced MaxEnt Model
Source: Insects. 2025 Mar 3;16(3):262. doi: 10.3390/insects16030262 (PMC11943047; doi:10.3390/insects16030262)
Supplement: Supplementary file 1 [file insects-16-00262-s001.zip › Supplementary Table S3.pdf]

Table S3 The optimize model parameters.

Name	Model	Omission_rate_at_5%	delta_AICc
Ophiocordyceps sinensis	M_0.8_F_qp_Set_1	0.044642857	0
host insects	M_0.5_F_lpt_Set_1	0.043165468	0
Chinese Caterpillar Fungus (CCFs)	M_0.8_F_lph_Set_1	0.03030303	0
