# Supplementary material for: Quantifying Potentially Suitable Geographical Habitat Changes in Chinese Caterpillar Fungus with Enhanced MaxEnt Model
Source: Insects. 2025 Mar 3;16(3):262. doi: 10.3390/insects16030262 (PMC11943047; doi:10.3390/insects16030262)
Supplement: Supplementary file 1 [file insects-16-00262-s001.zip › Supplementary Table S4.pdf]

Table S4 Percentage contribution of 7 environment variables of the *O. sinensis*.

Variable	Description	Percent contribution (%)	Permutation importance
Elev	Altitude (elevation above sea level) (m)	39.6	60.1
Slope	Slope	1.1	0
Bio3	Isothermality (BIO2/BIO7) (×100)	3	5.7
Bio9	Mean Temperature of Driest Quarter	15.8	25.4
Bio14	Precipitation of Driest Month	3.9	4.9
Bio15	Precipitation Seasonality (Coefficient of Variation)	8.2	0.3
Bio18	Precipitation of Warmest Quarter	28.3	3.5
