# Supplementary material for: Quantifying Potentially Suitable Geographical Habitat Changes in Chinese Caterpillar Fungus with Enhanced MaxEnt Model
Source: Insects. 2025 Mar 3;16(3):262. doi: 10.3390/insects16030262 (PMC11943047; doi:10.3390/insects16030262)
Supplement: Supplementary file 1 [file insects-16-00262-s001.zip › Supplementary Table S5.pdf]

**Table S5 Percentage contribution of 7 environment variables of host insects.**

| Variable | Description | Percent contribution (%) | Permutation importance |
|---|---|---|---|
| Elev | Altitude (elevation above sea level) (m) | 43.6 | 81.2 |
| Slope | Slope | 21.7 | 1 |
| Bio3 | Isothermality (BIO2/BIO7) (×100) | 9.8 | 2.4 |
| Bio8 | Mean Temperature of Wettest Quarter | 1.1 | 1.1 |
| Bio9 | Mean Temperature of Driest Quarter | 5.8 | 3.2 |
| Bio15 | Precipitation Seasonality (Coefficient of Variation) | 0.8 | 2.1 |
| Bio18 | Precipitation of Warmest Quarter | 17.1 | 9 |
