# Supplementary material for: Quantifying Potentially Suitable Geographical Habitat Changes in Chinese Caterpillar Fungus with Enhanced MaxEnt Model
Source: Insects. 2025 Mar 3;16(3):262. doi: 10.3390/insects16030262 (PMC11943047; doi:10.3390/insects16030262)
Supplement: Supplementary file 1 [file insects-16-00262-s001.zip › Supplementary Table S6.pdf]

Table S6 Percentage contribution of 6 environment variables of the Chinese Caterpillar Fungus (CCFs).

Variable	Description	Percent contribution (%)	Permutation importance
Elev	Altitude (elevation above sea level) (m)	49.3	84.8
Slope	Slope	23.2	0.6
Bio8	Mean Temperature of Wettest Quarter	1.5	0.9
Bio9	Mean Temperature of Driest Quarter	0.9	4
Bio15	Precipitation Seasonality (Coefficient of Variation)	1	0.5
Bio18	Precipitation of Warmest Quarter	24	9.1
