# Supplementary material for: Quantifying Potentially Suitable Geographical Habitat Changes in Chinese Caterpillar Fungus with Enhanced MaxEnt Model
Source: Insects. 2025 Mar 3;16(3):262. doi: 10.3390/insects16030262 (PMC11943047; doi:10.3390/insects16030262)
Supplement: Supplementary file 1 [file insects-16-00262-s001.zip › Supplementary Table S7.pdf]

Table S7 Current areas of habitat distribution for *Ophiocordyceps sinensis*, host insects, and the Chinese Caterpillar Fungus

Species	Low Habitat Suitability （$\times 10^4$ km^2）	Medium Habitat Suitability （$\times 10^4$ km^2）	High Habitat Suitability （$\times 10^4$ km^2）	Unsuitable habitat （$\times 10^4$ km^2）	Percentage of Highly Suitable Areas in the Total Area of China （%）	Percentage of Medium-suitable Areas in the Total Area of China(%)
Ophiocordyceps sinensis	93.75	25.19	74.28	767.85	7.73	2.62
host insects	47.48	36.25	66.63	810.70	6.93	3.77
both O. sinensis and host insects to coexist	/	/	56.87	877.03	7.08	/
Chinese Caterpillar Fungus	52.39	29.28	64.06	815.35	6.67	3.05
