# Supplementary material for: Quantifying Potentially Suitable Geographical Habitat Changes in Chinese Caterpillar Fungus with Enhanced MaxEnt Model
Source: Insects. 2025 Mar 3;16(3):262. doi: 10.3390/insects16030262 (PMC11943047; doi:10.3390/insects16030262)
Supplement: Supplementary file 1 [file insects-16-00262-s001.zip › Supplementary Table S8.pdf]

Table S8 Analysis of the highly suitable distribution areas for *O. sinensis*.

Province	Highly Suitable Area (10^4 km^2)	Total ($\times 10^4$ km^2)	Percentage of Highly Suitable Area in Province (%)	Percentage of Highly Suitable Areas in China (%)
Gansu	2.68	42.59	6.29	0.28
Qinghai	10.80	72.1	14.98	1.12
Tibet	36.47	122.84	29.69	3.79
Sichuan	22.80	48.6	46.91	2.37
Yunnan	1.52	39.4	3.87	0.16
Guizhou	0.01	17.62	0.04	-
China	74.28	/	/	7.73
