# Supplementary material for: Quantifying Potentially Suitable Geographical Habitat Changes in Chinese Caterpillar Fungus with Enhanced MaxEnt Model
Source: Insects. 2025 Mar 3;16(3):262. doi: 10.3390/insects16030262 (PMC11943047; doi:10.3390/insects16030262)
Supplement: Supplementary file 1 [file insects-16-00262-s001.zip › Supplementary Table S9.pdf]

Table S9 Analysis of the highly suitable distribution areas for host insects.

Province	Highly Suitable Area ($\times 10^4$ km^2)	Total ($\times 10^4 km^2$)	Percentage of Highly Suitable Area in Province	Percentage of Highly Suitable Areas in China
Gansu	3.69	42.59	8.65	0.38
Qinghai	12.36	72.1	17.15	1.29
Tibet	25.71	122.84	20.93	2.67
Sichuan	21.95	48.6	45.17	2.28
Yunnan	2.90	39.4	7.39	0.30
Taiwan	0.02	3.6	0.43	/
China	66.63	/	/	6.93
