# Supplementary figures and images for: Quantifying Potentially Suitable Geographical Habitat Changes in Chinese Caterpillar Fungus with Enhanced MaxEnt Model
Source: Insects. 2025 Mar 3;16(3):262. doi: 10.3390/insects16030262 (PMC11943047; doi:10.3390/insects16030262)

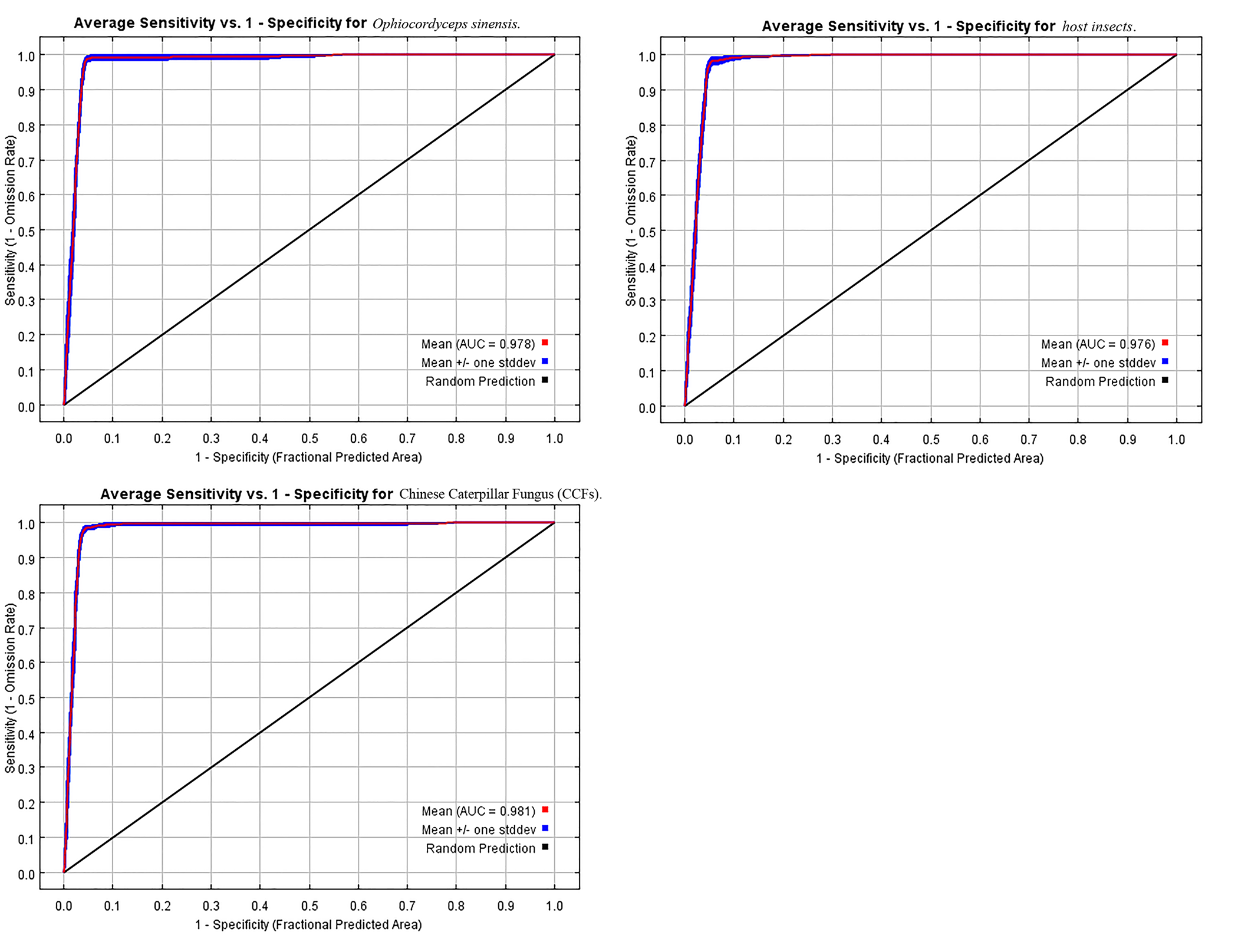

Supplement: Supplementary file 1 [file insects-16-00262-s001.zip › Figure S1.tif]

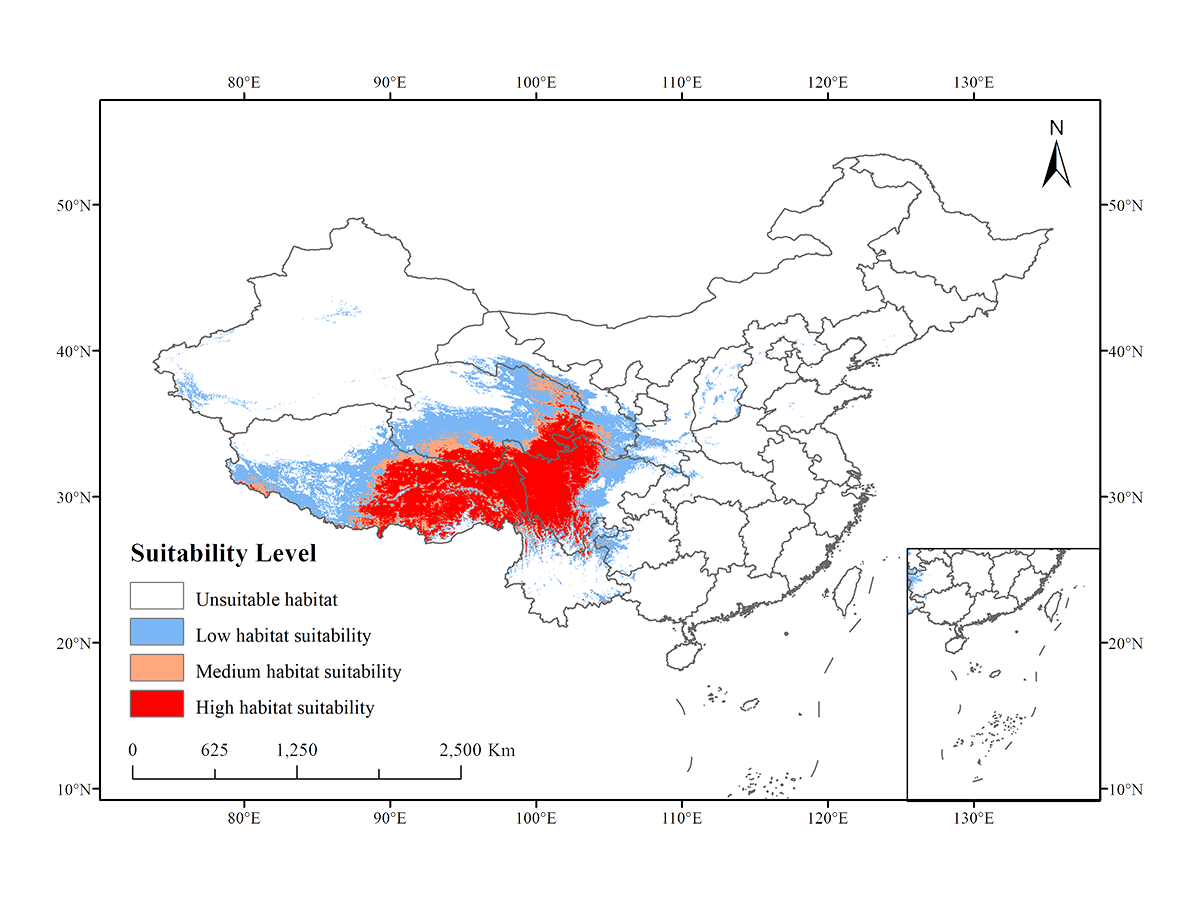

Supplement: Supplementary file 1 [file insects-16-00262-s001.zip › Figure S2.tif]

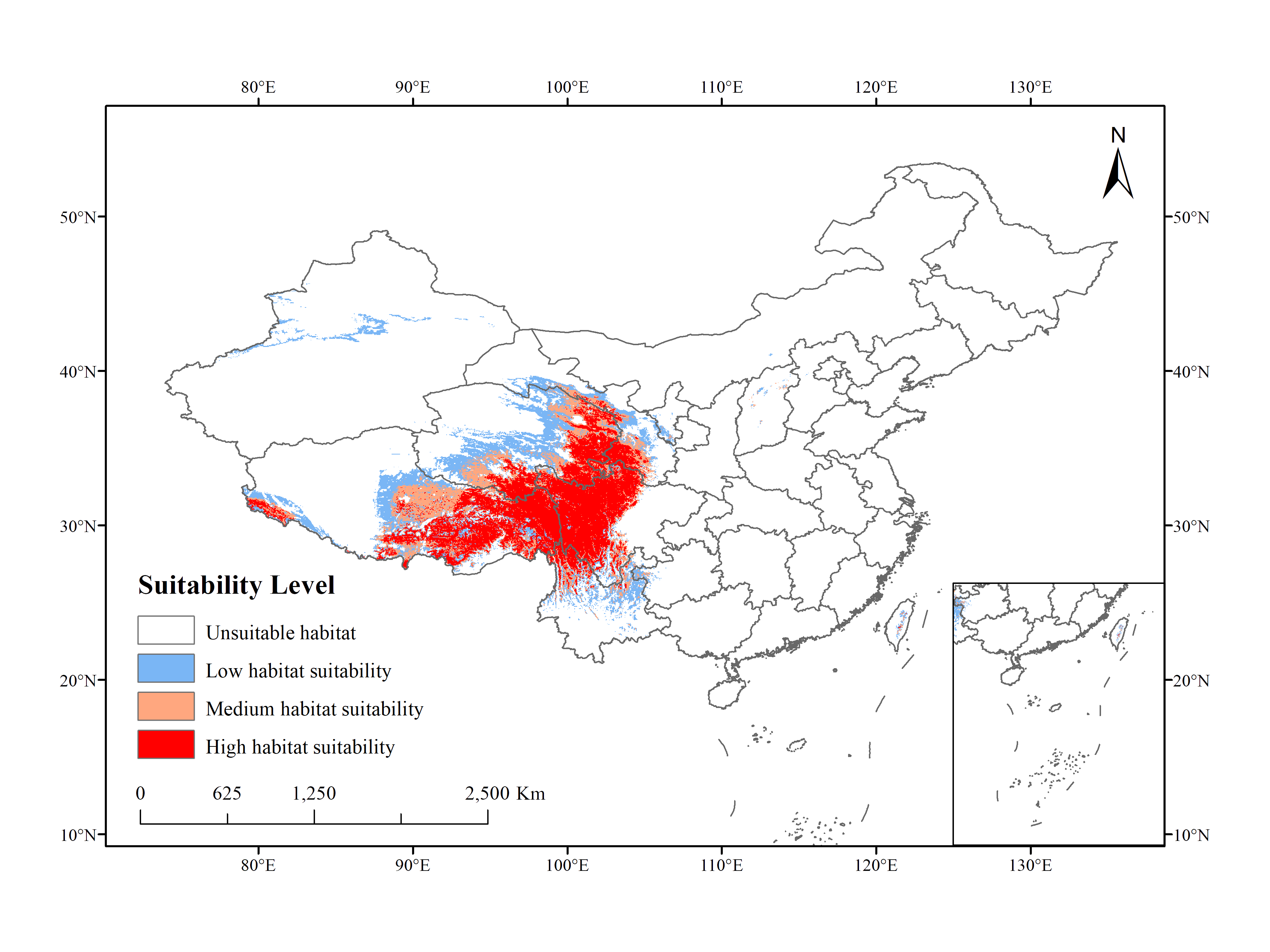

Supplement: Supplementary file 1 [file insects-16-00262-s001.zip › Figure S3.tif]

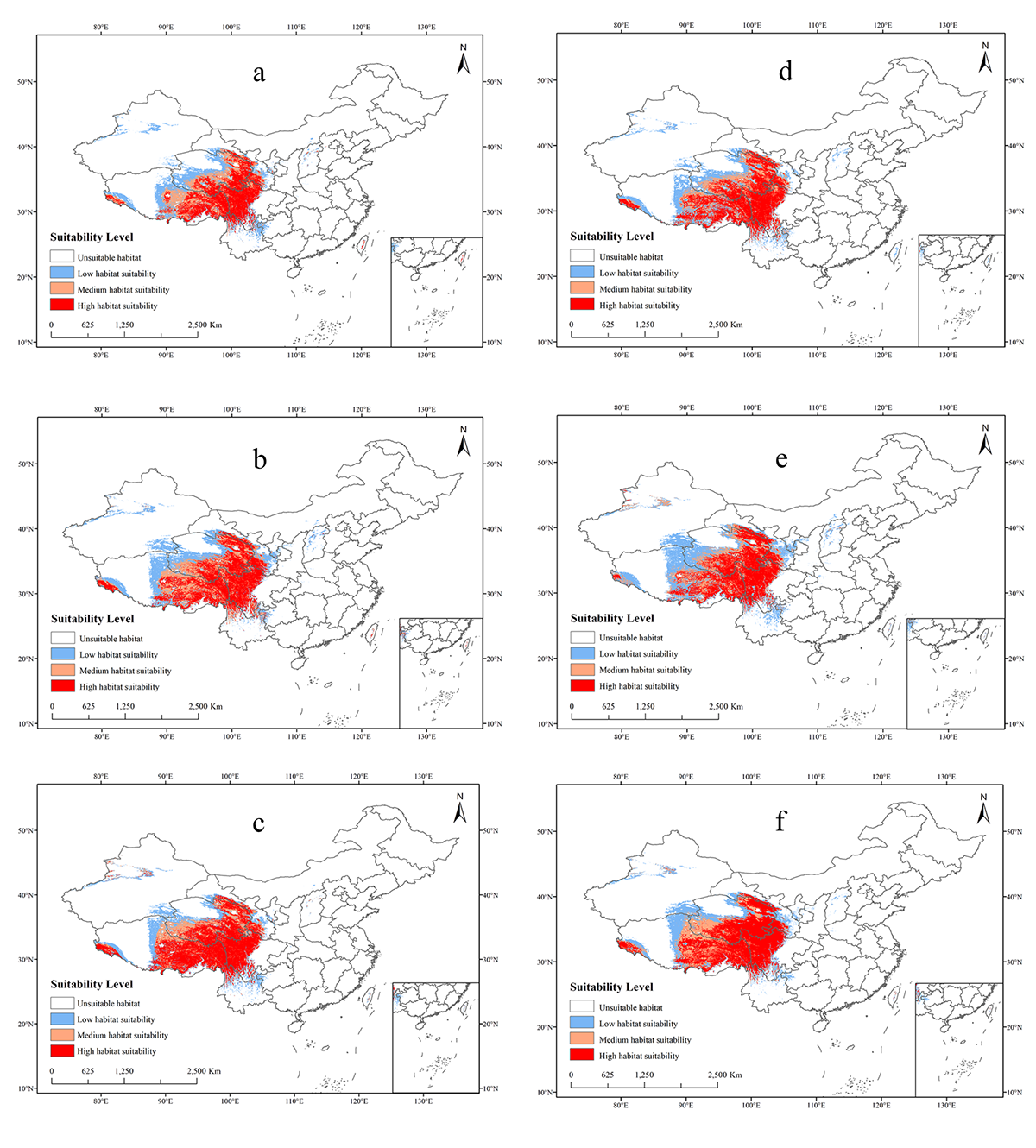

Supplement: Supplementary file 1 [file insects-16-00262-s001.zip › Figure S4.tif]

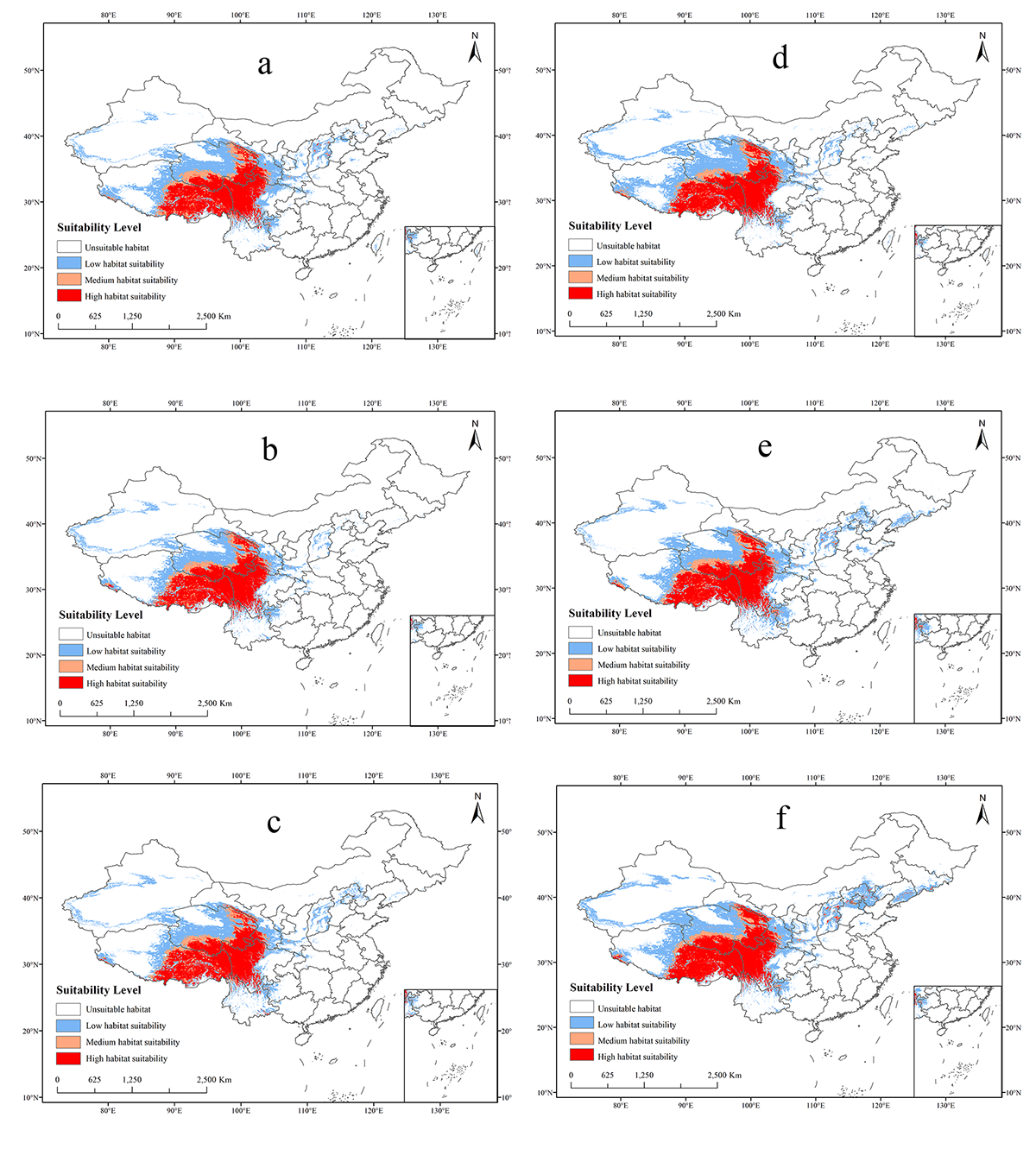

Supplement: Supplementary file 1 [file insects-16-00262-s001.zip › Figure S5.tif]
